# Experimental Study on Flexural Performance of SFCB-Reinforced ECC-Concrete Composite Beams

**DOI:** 10.3390/polym17202794

**Published:** 2025-10-19

**Authors:** Yu Ling, Shuo Xu, Chaohao Bi, Zile Feng, Dian Liang, Yongjian Cai

**Affiliations:** 1 Guangzhou Power Supply Bureau, Guangdong Power Grid Co., Ltd., China Southern Power Grid Co., Ltd., Guangzhou 510665, China; 2 School of Civil and Transportation Engineering, Guangdong University of Technology, Guangzhou 510006, China; 3 Guangdong Xiangtai Inspection and Appraisal Co., Ltd., Guangzhou 510000, China

**Keywords:** steel-FRP composite bars (SFCBs), engineered cementitious composites (ECC), composite beams, cracking behavior, flexural behavior

## Abstract

Engineered Cementitious Composite (ECC) exhibits superior tensile strain-hardening behavior and enhanced crack control due to its distinctive multiple cracking characteristic. In contrast, Steel–Glass Fiber Reinforced Polymer (GFRP) Composite Bars (SFCBs) combine the ductility of steel with the corrosion resistance of GFRP. To investigate the synergistic mechanisms for optimizing the performance of concrete structures, this study designed eight SFCB-reinforced ECC-concrete composite beams. Four-point bending tests were conducted to examine the influence of the ECC replacement height in the tension zone (hE/h = 0%, 16.67%, 33.33%, 50%) and the steel ratio in the bottom longitudinal reinforcement (As/Ab = 0%, 9%, 25%, 49%, 100%) on the flexural performance. The experimental results demonstrated the following: (1) Increasing the ECC replacement significantly improved both the ultimate bending capacity and ductility, while exerting a limited effect on flexural stiffness. Specifically, when increased from 0% to 50%, the ultimate bending strength and ductility index increased by 4.79% and 8.09%, respectively. (2) The steel ratio predominantly governed the yield behavior and crack development. Higher steel ratios resulted in increased flexural stiffness prior to yield, higher yield moments, improved ductility at failure, and superior crack control capability before yielding. (3) The synergistic mechanisms were identified: the ECC layer optimizes crack control by distributing crack-induced strains through multiple cracking, while the steel ratio within the SFCB regulates the ductile response. The findings of this study provide valuable theoretical guidance for enhancing the capacity and ductility of building structures.

## 1. Introduction

Reinforced concrete structures owing to the complementary material advantages of concrete in compression and steel reinforcement in tension, have become the predominant structural form in the construction industry [1,2,3]. However, with continuously escalating performance demands for building structures, conventional reinforced concrete faces significant challenges in load-carrying capacity, durability, and ductility [4,5]. Consequently, researchers persistently pursue innovative materials and structural systems to enhance the mechanical performance and service life of building structures [6,7].

Although conventional concrete possesses excellent compressive strength, its low tensile strength, pronounced post-cracking softening behavior, and tendency for rapid crack propagation result in insufficient structural ductility and vulnerability to environmental degradation [6]. Engineered Cementitious Composite (ECC), pioneered by Victor C. Li [8,9], is an ultra-ductile cement-based material. Leveraging fiber bridging mechanisms, ECC transforms concrete cracking behavior from localized macro-cracking to distributed micro-cracking [10,11,12], maintaining significant load-bearing capacity after cracking and thereby overcoming the tensile softening inherent in conventional concrete [13,14]. Consequently, ECC exhibits superior tensile strain-hardening behavior and exceptional crack control capabilities, offering an effective solution to mitigate the inherent brittleness and excessive crack widths of conventional concrete structures [15,16,17].

Steel reinforcement serving as the primary reinforcement in conventional concrete, provides essential structural ductility. Nevertheless, it is susceptible to corrosion in aggressive environments such as deicing salt applications or marine exposure, compromising structural durability. Over the past two decades, replacing steel with lightweight, high-strength, and corrosion-resistant Fiber-Reinforced Polymer (FRP) bars has gained considerable research interest, with cost-effective Glass FRP (GFRP) bars receiving particular attention [18,19,20]. However, due to the linear-elastic tensile behavior and lower elastic modulus of GFRP bars, GFRP bar-reinforced concrete structures exhibit brittle failure modes, excessive deflections, and wider cracks compared to their steel-reinforced counterparts [21]. To address this limitation, Steel-FRP Composite Bars (SFCBs) have been proposed, featuring a steel core for ductility encased within a protective FRP shell for corrosion resistance [22,23]. SFCBs synergistically combine the ductility of steel with the corrosion resistance of FRP [24,25,26,27,28,29], presenting a novel approach to enhance both the ductility and durability of concrete structures.

Collectively, compared to traditional reinforced concrete, SFCB-reinforced ECC structures demonstrate significant advantages in mechanical performance and durability. However, the high cost of ECC, approximately four times that of conventional concrete [30], makes full replacement economically unfeasible. A practical strategy involves utilizing the tensile advantages of ECC by partially replacing conventional concrete solely within the tension zone [31,32,33]. Yuan et al. [31] demonstrated that FRP bar-reinforced ECC-concrete composite beams exhibit superior energy dissipation capacity relative to conventional reinforced concrete beams. Ge et al. [32] investigated the effects of reinforcement ratio, reinforcement type, and ECC replacement height (*h_E_*/*h*) on the flexural performance of composite beams. They found that replacing concrete with ECC of identical thickness in the tension zone enhanced flexural capacity, stiffness, and crack control effectiveness; the degree of enhancement depended significantly on the type and amount of reinforcement. Qin et al. [33] further reported that steel bar-reinforced composite beams featuring ECC of varying thickness and configuration exhibited superior ductility and energy absorption capacity. These studies collectively validate the effectiveness of the ECC-concrete composite form in enhancing structural mechanical performance and crack control. Furthermore, beyond the improved tensile strain-hardening and crack control capabilities discussed herein, ECC has also demonstrated superior performance in mitigating long-term durability issues such as creep, fatigue crack propagation, and corrosion-induced deterioration due to its tight crack width and intrinsic material properties [34,35,36,37]. This makes it particularly suitable for synergistic use with corrosion-resistant SFCBs. While the aforementioned studies have provided valuable insights into the behavior of FRP-reinforced or steel-reinforced ECC-concrete beams, investigations on the flexural performance of beams reinforced withSFCBs, which synergistically combine the ductility of steel and the corrosion resistance of FRP-remain scarce. Notably, recent advanced studies have primarily focused on composite beams reinforced with either pure steel or pure FRP bars [38]. However, a systematic experimental investigation into the synergistic effects of SFCBs and ECC in a composite beam system, particularly evaluating the interplay between the ECC replacement height and the critical steel ratio within the SFCB, has not been previously undertaken. This study aims to fill this research gap by systematically evaluating the synergistic effects of SFCBs and ECC in a composite beam system.

The tensile stress–strain relationship of SFCBs exhibits a distinct bilinear profile [23,39]. Compared to pure FRP bars, SFCBs display a more pronounced yield point and higher elastic modulus, while offering superior corrosion resistance and a stable post-yield hardening modulus compared to pure steel bars [40,41]. Consequently, the flexural performance of SFCB-reinforced concrete beams differs fundamentally from that of beams reinforced with steel or FRP bars alone. Lin et al. [42] confirmed that the flexural behavior of SFCB-reinforced concrete beams depends critically on the FRP-to-steel cross-sectional area ratio (*A_f_*/*A_s_*). Under identical total reinforcement ratios, a higher FRP proportion reduced the yield capacity and ductility of the beams. Specifically, when the FRP layer proportion was 0%, beam behavior approached that of conventional steel bar-reinforced concrete; at 100%, it converged towards the behavior of FRP bar-reinforced concrete beams. Han et al. [43], after equating the FRP layer contribution to stiffness-equivalent steel for testing purposes, observed that SFCB-reinforced concrete beams exhibited flexural behavior similar to steel bar-reinforced beams prior to yielding under equivalent reinforcement ratios, but achieved higher ultimate capacity and deformability after yielding. These findings underscore the significant influence of the steel core-to-FRP proportion within SFCBs on structural mechanics, highlighting the critical need for research into the flexural performance of SFCB-reinforced ECC-concrete composite beams.

In recent years, Digital Image Correlation (DIC) technology has become a powerful tool for evaluating deformation and crack development in concrete structures. By analyzing image sequences to measure full-field displacement and strain, DIC provides high-resolution data that is particularly suitable for studying the flexural and shear performance of reinforced concrete beams. For example, Aksoylu et al. [44] used the DIC method to assess the bending performance of RC beams with waste glass powder (WGP) replacing cement, demonstrating the effectiveness of DIC in real-time crack detection. Similarly, Karalar et al. [45] compared the shear performance of RC beams containing recycled glass powder and utilized DIC for crack analysis and failure mode validation. Furthermore, Zeybek et al. [46] analyzed the shear behavior of beams with waste glass as aggregate replacement through DIC and theoretical methods, highlighting the advantage of DIC in providing precise strain data. These studies not only demonstrate the reliability of DIC technology but also provide a solid foundation for the adoption of the DIC method in this research, particularly in the application of sustainable materials such as waste glass in concrete.

Therefore, this study designed and tested eight SFCB-reinforced ECC-concrete composite beams with varying parameters. Utilizing four-point bending tests, the research systematically investigates the influence of ECC replacement height in the tension zone (*h_E_*/*h* = 0%, 16.67%, 33.33%, 50%) and the steel ratio within the bottom longitudinal reinforcement (*A_s_*/*A_b_* = 0%, 9%, 25%, 49%, 100%) on the flexural performance. The results aim to provide fundamental reference for subsequent research and engineering design.

## 2. Materials and Methods

### 2.1. Materials

This section details the constituent materials of the composite beams: conventional concrete, ECC, and reinforcement bars. C40 grade commercial concrete, supplied by Guangzhou Jinjian Building Materials Co., Ltd. (Guangzhou, China), was used for the conventional concrete. During beam casting, following Chinese standard GB/T 50081-2019 [47], three 150 mm cubes and three φ 150 mm × 300 mm cylinders were fabricated. After 28-day curing, mechanical compression tests yielded the following properties for the conventional concrete: average cube compressive strength = 46.72 MPa, average cylinder compressive strength = 42.53 MPa, elastic modulus = 33.85 GPa, and Poisson’s ratio = 0.22.

The ECC mixture employed in this study was modified from established formulations [10,48,49]. The ECC consisted of cement, fly ash (FA), quartz powder (QP), water, superplasticizer, PE fibers, viscosity modifying agent (VMA), and defoamer. The mix proportions are provided in Table 1. Ordinary Portland cement (Grade 52.5 R) was used. FA was Class F Grade II. Quartz powder (particle size: 50–300 μm) served as the fine aggregate. A high-performance polycarboxylate ether-based superplasticizer was used to enhance workability. Hydroxypropyl methylcellulose (HPMC), a milky-white powder, acted as the viscosity modifier to improve fiber dispersion. The defoamer, a mixture of liquid hydrocarbons, polyoxyethylene glycol, and amorphous silicon dioxide (active content: 65%), minimized entrapped air bubbles during mixing to reduce internal defects. Ultra-high molecular weight polyethylene (UHMWPE) fibers, providing the primary bridging mechanism, were incorporated due to their high modulus and toughness. Manufacturer-supplied fiber parameters are detailed in Table 2.

To ensure uniform fiber dispersion, a key factor influencing crack distribution, hydroxypropyl methylcellulose (HPMC) was used as a viscosity modifying agent. The mixing procedure was strictly controlled: dry components (cement, FA, QP) were mixed for 3 min, followed by the gradual addition of water containing dissolved superplasticizer and HPMC. Finally, PE fibers were added slowly and mixed for an additional 5 min until a homogeneous distribution was achieved. It exhibited a slump of 180 mm, satisfying the workability requirements for beam casting. While the mixing procedure was optimized to ensure uniform fiber dispersion, this study acknowledges a fundamental limitation: controlling and quantifying the in situ orientation of fibers during the casting of full-scale beam specimens presents considerable practical challenges. The complex flow of the fresh ECC mixture during placement inevitably influences the final fiber orientation distribution, which may contribute to the scatter in local cracking behavior and mechanical test results.

**Figure 1:**
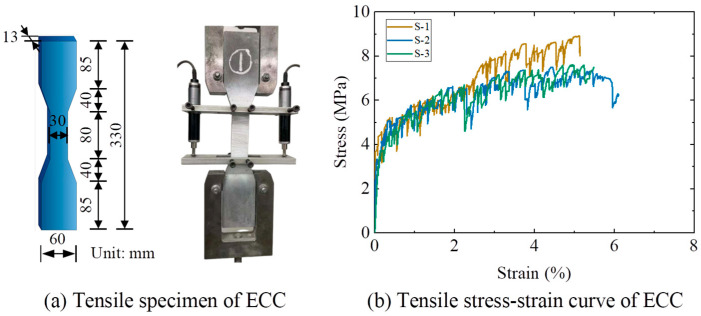
Tensile test of ECC.

Following Chinese standard JC/T 2461-2018 [50], the tensile mechanical properties of ECC were determined using dumbbell-shaped specimens (330 mm × 60 mm × 13 mm), as illustrated in Figure 1. Tensile tests indicated average first-cracking stress and strain values of 2.89 MPa and 0.0004, respectively, and average ultimate tensile stress and strain values of 8.04 MPa and 0.055, respectively.

**Figure 2:**
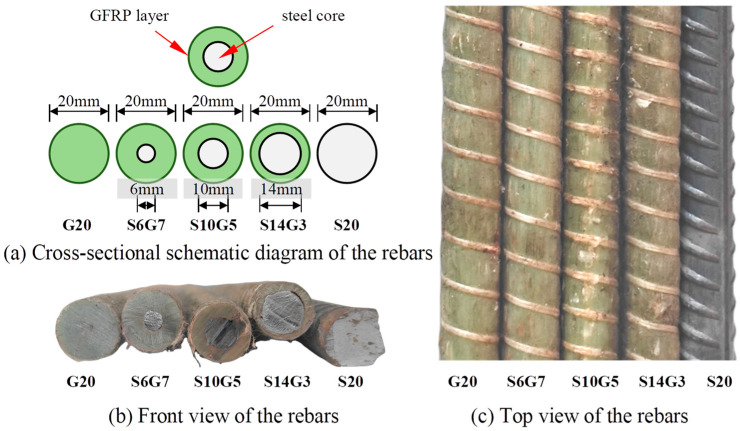
Diagram of rebars.

**Figure 3:**
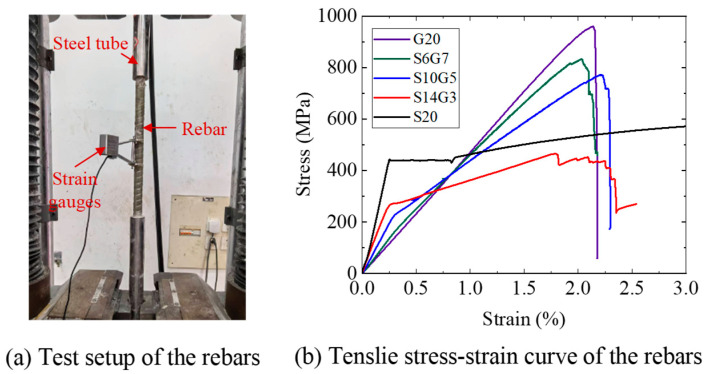
Tensile test of the rebars.

Five types of rebars, all 20 mm in nominal diameter, were used as the bottom longitudinal reinforcement in the composite beams, as shown in Figure 2. These comprised three Steel-GFRP Composite Bars (SFCBs), one pure GFRP bar, and one steel bar, all supplied by Shenzhen Haichuan New Materials Technology Co., Ltd. (Shenzhen, China) The SFCBs featured HRB400 steel cores with diameters of 6 mm, 10 mm, and 14 mm, encapsulated by GFRP layer with thicknesses of 7 mm, 5 mm, and 3 mm, respectively. The pure GFRP bar had a nominal diameter of 20 mm, and exhibited identical material properties to the GFRP layer of SFCBs. The pure steel bar was a 20 mm diameter HRB400 steel bar. Tensile tests on the rebars were conducted in accordance with GB/T 30022-2013 [51], refer to Figure 3. GFRP is anisotropic, meaning its shear strength is much lower than its tensile strength [52]. To prevent the bar ends from failing during testing, we reinforced them with seamless steel tubes bonded using expansive cement mortar. Detailed mechanical properties of all reinforcement bars are presented in Figure 3b and Table 3.

### 2.2. Specimen Design

**Figure 4:**
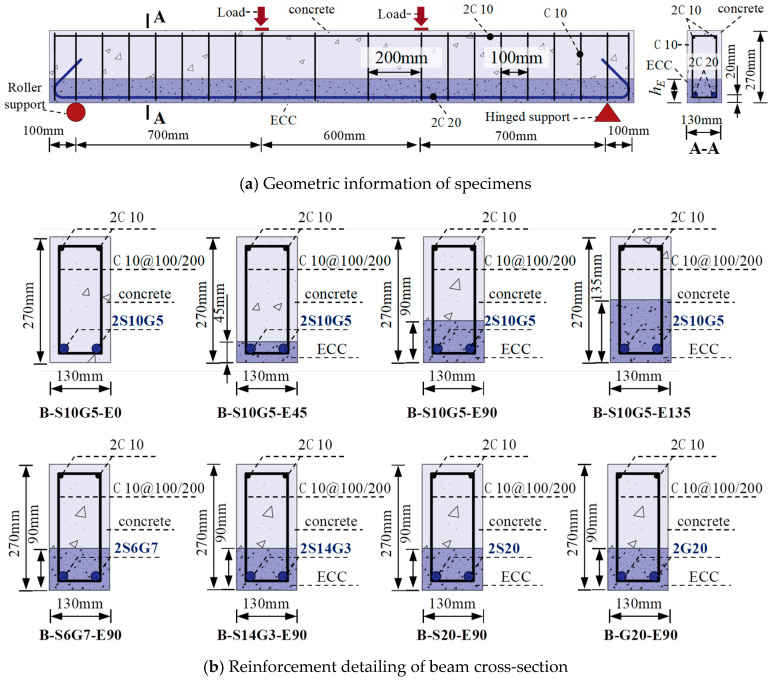
Specimen design.

To investigate the flexural behavior of SFCBs reinforced ECC-concrete composite beams, eight specimens were designed and tested under four-point bending, as shown in Figure 4. A parametric study was employed to examine the influence of both ECC replacement height within the tension zone and the type of bottom longitudinal reinforcement on the bending behavior. As illustrated in Figure 4a, the cross-sectional dimensions of the beams were b × h = 130 mm × 270 mm, with a total length lof 2200 mm and a clear span *l_o_* of 2000 mm. The pure bending zone spanned 600 mm, flanked by two shear-bending zones of 700 mm each. The top longitudinal reinforcement consisted of two ϕ 10 mm HRB400 bars. Stirrups, made of ϕ 10 mm HRB400 bars, were spaced at 100 mm within the shear-bending zones and at 200 mm within the pure bending zone to prevent shear failure. Following the recommendations of standards ACI 440.1R-2015 [53] and ACI 318-2019 [54], the beams were designed as under-reinforced. The bottom reinforcement comprised two longitudinal bars with a nominal diameter of 20 mm, resulting in a reinforcement ratio of 1.79%.

Reinforcement details for all specimens are shown in Figure 4b and listed in Table 4. Specimens were divided into two groups based on ECC replacement height and bottom reinforcement type. Group I specimens employed identical bottom reinforcement (i.e., two S10G5) but varying ECC replacement heights: *h_E_* = 0 mm, 45 mm, 90 mm, and 135 mm, corresponding to ECC replacement ratios (*h_E_*/*h*) of 0%, 16.67%, 33.33%, and 50%, respectively. The upper limit of 50% was selected based on beam theory to ensure the ECC layer remained within the tensile zone, thereby optimizing its strain-hardening capabilities and preventing material waste in the compression zone. Group II aimed to analyze the influence of the reinforcement type and steel ratio. These specimens featured a constant ECC replacement height (*h_E_*/*h* = 90 mm) but different bottom reinforcement types: G20 (pure GFRP bar), S6G7, S10G5, S14G3 (SFCBs), and S20 (pure steel bar). This selection resulted in steel ratios (*A_s_*/*A_b_*) of 0%, 9%, 25%, 49%, and 100%, respectively, enabling a comprehensive investigation into the transition from FRP-dominated to steel-dominated structural behavior while maintaining practical relevance.

### 2.3. Test Setup

**Figure 5:**
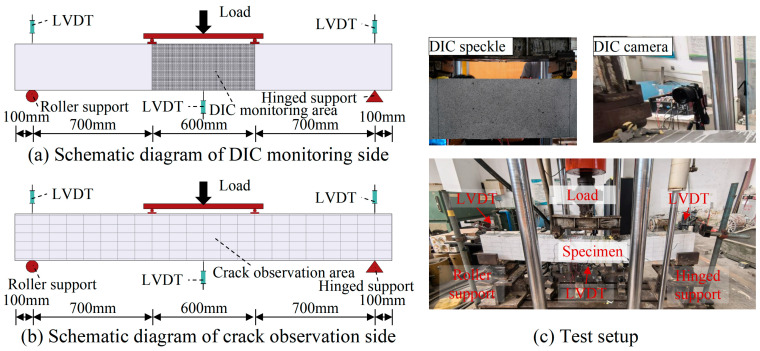
Test setup of four-point bending test.

All composite beams were tested under four-point bending using a 500 kN electro-hydraulic servo-controlled testing machine (Changchun Machine, Changchun, China), as shown in Figure 5. The load was applied uniformly to two loading points via a spreader beam. The distance between each support and its adjacent loading point was 700 mm, resulting in a pure bending span of 600 mm between the loading points. Three Linear Variable Displacement Transducers (LVDTs) were positioned at the supports and midspan to measure beam deflection. Two strain gauges were attached to the midspan region of each bottom longitudinal bar to measure reinforcement deformation. As depicted in Figure 5a,b, Digital Image Correlation (DIC) was employed to monitor real-time crack propagation on the side face of the pure bending segment. A DIC system consisting of a SONY a7R3 camera (Sony Corp., Tokyo, Japan; 7952 × 5304 pixels) was used to monitor crack propagation on the side face of the pure bending segment (600 mm × 270 mm). This setup provided a spatial resolution of approximately 0.075 mm/pixel. Images were acquired at a rate of 1 frame per second, which was suitable for capturing crack evolution under the quasi-static loading conditions (1 mm/min) employed in this study. The displacement measurement accuracy of the DIC system was validated by comparing the mid-span vertical displacement with readings from a calibrated LVDT. The results showed excellent agreement, confirming the reliability of the DIC measurements for this study. The images were processed using PMLABTM software (2019 Educational Edition) to obtain full-field displacement and strain contours. Strain fields were post-processed with a Gaussian filter (5 × 5 kernel) to reduce noise while preserving crack morphology. Additionally, a 100 mm × 50 mm grid was drawn on the opposite side of the beam to visually track overall crack development during testing. The tests were conducted under displacement control at a loading rate of 1 mm/min and terminated upon beam failure.

## 3. Results and Discussion

### 3.1. Failure Modes

**Figure 6:**
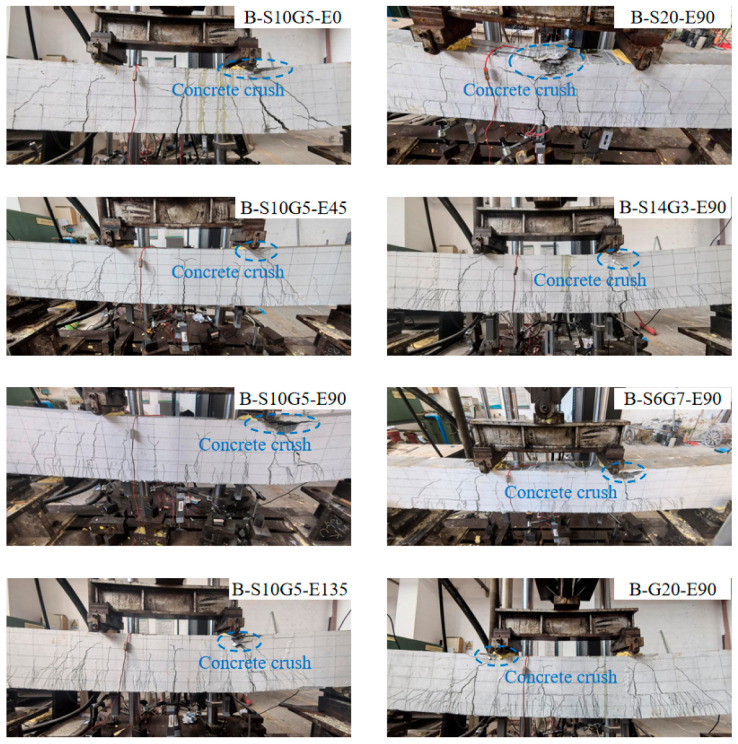
Failure modes of the composite beams.

Failure modes of the beams under four-point bending are shown in Figure 6. All specimens failed in a ductile, under-reinforced flexural mode, characterized by crushing of the concrete in the compression zone. Specifically, for beams reinforced with steel bars or SFCBs, failure occurred following yielding of the bottom longitudinal reinforcement and subsequent concrete crushing. Conversely, GFRP bar-reinforced composite beams failed by concrete crushing without rupture of the GFRP bars. These failure modes confirm that all composite beams performed as designed, avoiding undesirable failure mechanisms such as shear or tensile rupture of rebars. Taking specimen B-S10G5-E0 (without ECC replacement) as an example: initial flexural cracks first appeared at the bottom of the midspan pure bending zone during early loading. With increasing load, the initial cracks propagated vertically upwards, while new flexural cracks developed in other regions. As loading continued further, diagonal cracks formed within the shear-bending zones. Due to adequate stirrups for shear resistance, the specimen ultimately failed in flexure via concrete crushing after steel yielding. Compared to B-S10G5-E0, specimens with ECC replacement in the tension zone but identical bottom reinforcement developed a denser network of finer cracks at the bottom of the shear-bending zones. These fine cracks distributed around the primary concrete cracks. The number of fine cracks within the replacement layer significantly increased with higher ECC replacement ratios (e.g., from *h_E_*/*h* = 0% to 50%), demonstrating the effectiveness of ECC in suppressing crack width and mitigating environmental degradation risk. Furthermore, under identical ECC replacement heights, beams with different bottom reinforcement types (steel bar, SFCBs, GFRP bar) exhibited highly similar crack propagation patterns and final failure modes, i.e., all concrete crushing failures. This indicates an effective synergistic interaction between the bottom reinforcement and the ECC layer, optimizing the mechanical advantages of the composite system.

### 3.2. Load–Deflection Curves

**Figure 7:**
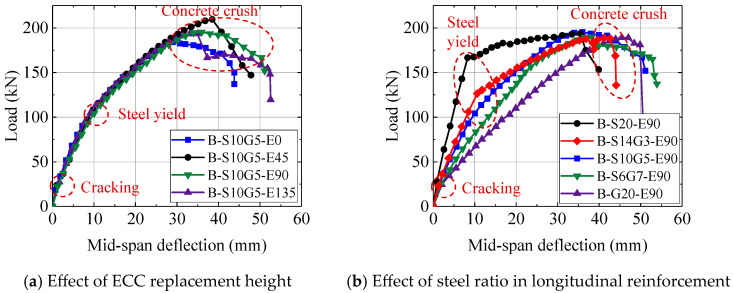
Load–deflection relationship of composite beam.

**Figure 8:**
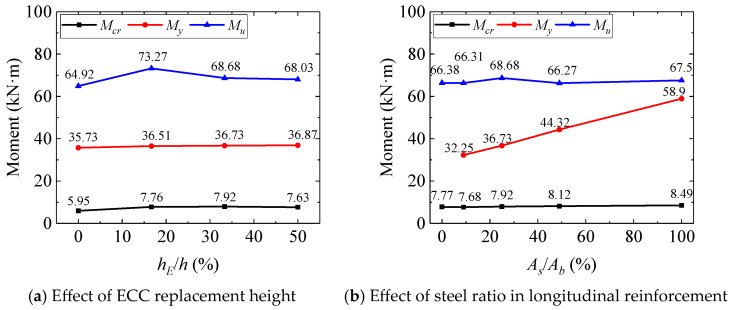
Bending moment of composite beam under different parameter.

Table 5 summarizes the test results from the four-point bending tests, while the load–deflection relationships of the composite beams are presented in Figure 7, and Figure 8 illustrates the variation in flexural capacity under different parameters.

Figure 7a reveals three distinct stages in the flexural behavior of SFCB-reinforced ECC-concrete composite beams under four-point bending. Stage I represents the uncracked state, where the bottom reinforcement, ECC, and concrete collectively resisted tensile stresses in the tension zone. Stage II commenced with cracking in the tension zone; subsequent tension redistribution away from cracked concrete to the reinforcement and ECC layer resulted in a significant reduction in stiffness. Stage III initiated upon yielding of the reinforcement, characterized by progressive stiffness degradation until concrete crushing failure. Notably, load–deflection curves for beams with increasing ECC replacement heights (*h_E_*/*h* from 0 mm to 135 mm) overlapped substantially. This suggests a marginal influence of ECC replacement on flexural stiffness, attributed to the limited contribution of the ECC layer to overall tension resistance compared to the dominant role of the bottom reinforcement [6]. Conversely, Figure 8a shows that ECC replacement significantly enhanced the ultimate flexural capacity (*M_u_*), despite minimal stiffness improvement. Increasing the replacement ratio from 0% to 16.67% (*h_E_* = 45 mm) increased *M_u_* by 12.86%. However, this gain diminished to 4.79% when the ratio reached 50% (*h_E_* = 135 mm). This non-linear enhancement likely stems from a synergistic mechanism: in thinner ECC layers, the confined casting space may restrict random fiber orientation, potentially promoting a higher degree of alignment along the loading direction. This hypothetical improvement in alignment could enhance fiber bridging efficiency, leading to denser micro-cracking. Conversely, as the structural size increases, the probability of encountering inherent material defects or areas of poor fiber dispersion also rises. Therefore, the contribution of the ECC replacement layer varies with scale effects. However, due to experimental cost constraints, this study only tested one specimen per parameter, precluding significance analysis of this effect. Future research should include statistical studies on ECC at different scales.

At lower replacement ratios, the fiber-bridging effect within the ECC layer delays crack coalescence, allowing concrete to better utilize its compressive potential; at higher ratios, the strength utilization efficiency of the thicker ECC layer decreases, resulting in diminishing marginal returns.

As shown in Figure 7b, the influence of bottom reinforcement type was pronounced under the same ECC replacement height. Higher steel ratios (*A_s_*/*A_b_*) resulted in greater post-cracking flexural stiffness. Due to their bilinear tensile behavior, beams reinforced with SFCBs exhibited a distinct yield plateau, unlike the GFRP bar-reinforced specimen (B-G20-E90). Compared to the steel bar-reinforced beam (B-S20-E90), SFCB-reinforced beams maintained a stable positive post-yield stiffness. Importantly, ultimate capacities (*M_u_*) were similar across all specimens, as shown in Table 5 or Figure 8b, indicating that the total reinforcement ratio primarily governs *M_u_*, which consistent with findings for SFCB-reinforced conventional concrete beams [42]. However, the steel ratio significantly regulated the yield moment (*M_y_*). As the steel ratio (*A_s_*/*A_b_*) increased from 9% (B-S10G5-E90) to 100% (B-S20-E90), *M_y_* increased by 82.6%. The mechanical characteristics of SFCBs thus offer a pathway for designing structures with enhanced toughness. By selecting an appropriate steel ratio for a given reinforcement area, both sufficient yield strength and robust post-yield recovery capacity can be achieved.

### 3.3. Load-Bottom Reinforcement Strain Relationship

**Figure 9:**
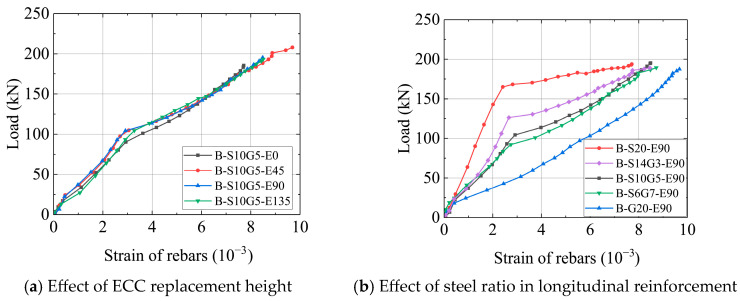
Load versus bottom reinforcement strain relationships.

Figure 9 shows the load versus bottom reinforcement strain relationships for the composite beams with different parameters.

As shown in Figure 9a, the strain development in the reinforcement followed a three-stage pattern irrespective of ECC replacement height. Stage I (Pre-cracking): Reinforcement strain remained low as the ECC layer (or concrete) shared the tensile load. Stage II (Post-cracking): Upon concrete cracking, tensile force was primarily transferred to the bottom reinforcement at cracked sections, leading to a significant increase in strain. Stage III (Post-yielding): After yielding of the steel core in the SFCBs, the reduced elastic modulus caused an accelerated rise in strain. Notably, the load-strain curves for different ECC replacement heights overlapped closely. This reconfirms that local ECC replacement does not alter the fundamental mechanics of tension resistance dominated by the reinforcement. It further emphasizes that the primary value of the ECC layer lies in crack control, not in sharing significant tensile load.

Figure 9b also shows that under identical ECC replacement and loading, bottom reinforcement with higher steel ratios (*A_s_*/*A_b_*) developed lower strains due to their greater load-carrying capacity per unit strain. This phenomenon provides a micromechanical explanation for the flexural stiffness differences observed in Figure 7b and offers theoretical support for toughness design: increasing the SFCB steel ratio reduces the damage (strain) level in critical components at a given load without increasing the total reinforcement ratio, thereby enhancing structural reparability.

### 3.4. Crack Development

**Figure 10:**
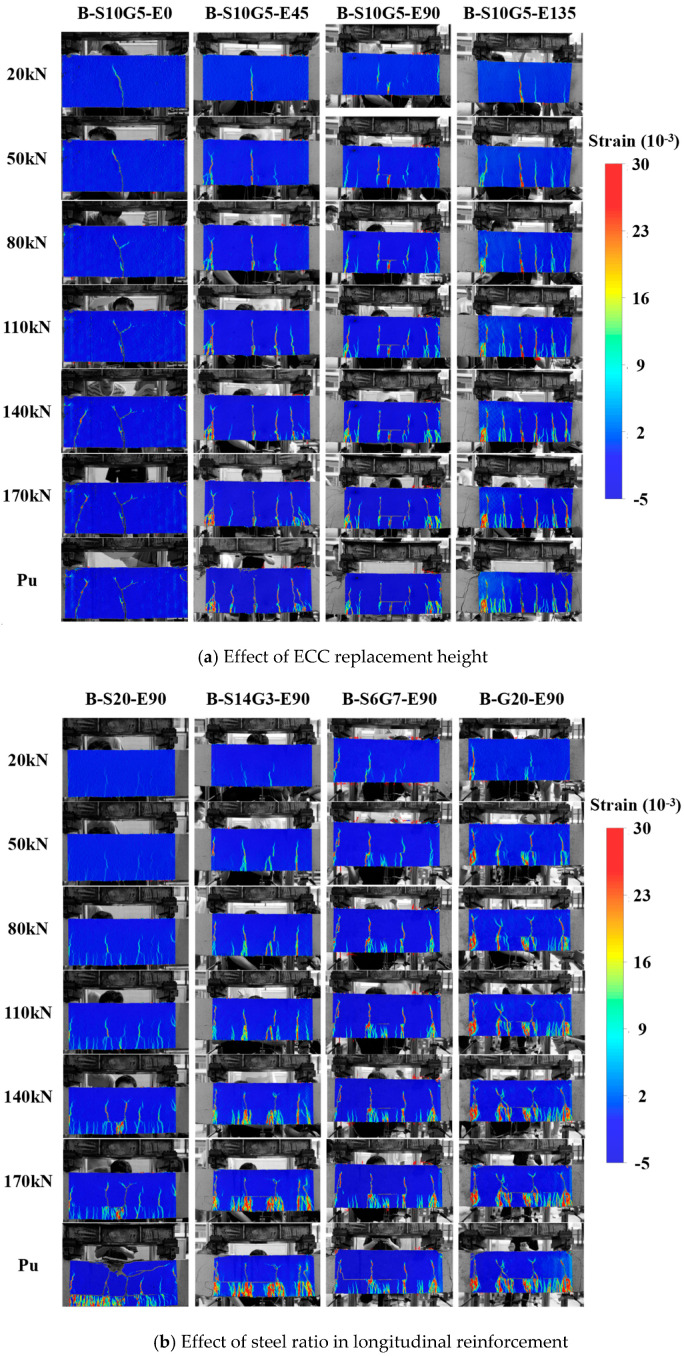
Crack development in pure bending zone.

Figure 10 displays DIC principal strain contour maps for the pure bending zone at different loading stages. Areas of concentrated tensile strain (warmer colors) reflect cracking behavior; regions approaching red indicate wider cracks.

Figure 10a shows that initial cracks formed at the beam soffit within the pure bending zone during early loading for all specimens. Compared to the non-ECC specimen B-S10G5-E0, which exhibited localized single-crack propagation, ECC-replaced specimens (B-S10G5-E45/E90/E135) generated significantly denser microcracks around the initial primary cracks during sustained loading. The distribution of high-strain areas indicates smaller crack widths and increased crack density with higher ECC replacement heights. This phenomenon arises from multiple cracking mechanism of ECC, where fiber bridging disperses macro-cracking into numerous fine microcracks, effectively controlling crack width.

Figure 10b further illustrates the influence of the steel ratio under the same ECC replacement height. At loads ≤ 110 kN, specimens with lower steel ratios (e.g., B-G20-E90, *A_s_*/*A_b_* = 0%) exhibited higher crack density and maximum tensile strain in the ECC layer, confirming that higher steel ratio reinforcement more effectively restrains early crack development. For loads > 110 kN, a reversal in crack density occurred: specimens with higher steel ratios developed more extensive cracking following reinforcement yielding and subsequent stiffness reduction. This multi-cracking morphology enhances energy absorption capacity, improving structural ductility. Therefore, designing for a moderate steel ratio offers a dual benefit: utilizing the high pre-yield stiffness to suppress crack initiation, while enabling a controlled, damage-tolerant ductile failure mode characterized by distributed cracking post-yield.

It should be noted that the current DIC analysis has several limitations: (1) The spatial resolution (0.075 mm/pixel) was sufficient for identifying macro-crack patterns and strain localization but near the limit for detecting the very initial formation of all ECC micro-cracks below 0.1 mm, making quantitative width measurement challenging; (2) DIC was applied only to one side of the beam, though visual inspection confirmed symmetric crack patterns; (3) The post-processing software did not allow for quantitative tracking of cumulative crack length or crack density evolution over loading history; (4) Quantitative extraction of maximum tensile strain values was constrained by software capabilities. While the qualitative DIC data provided valuable insights into crack distribution patterns and strain fields, future studies should employ higher-resolution cameras, 3D-DIC systems, or advanced software tools to enable more precise quantitative analysis of micro-crack characteristics and evolutionary parameters.

### 3.5. Ductility of Composite Beams

**Figure 11:**
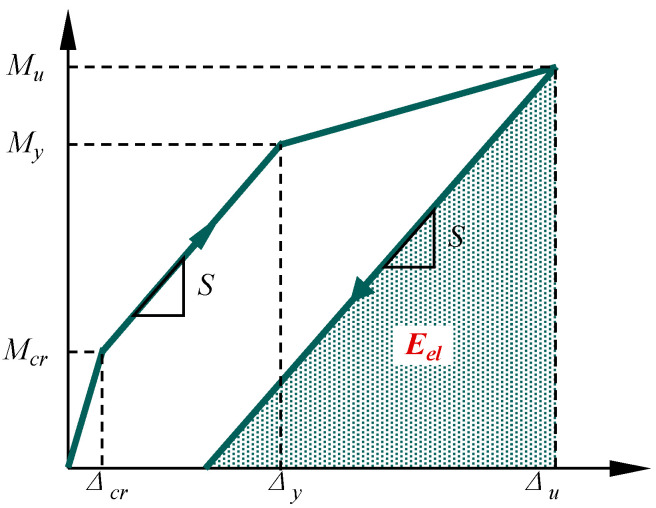
Energy dissipation-based ductility index.

Ductility is a key metric for evaluating the flexural performance of concrete beams. Conventionally, it is quantified as the ratio of ultimate deflection to yield deflection (*μ* = *Δ_u_*/*Δ_y_*. However, this method is unsuitable for beams reinforced with non-yielding GFRP bars. Consequently, Oudah and Nemati [55] proposed an energy-based ductility index, defined as:

(1) $$\mu =\frac{E_{tol}}{E_{el}}$$

where *E_tol_* is the total energy absorbed until failure, calculated as the area under the moment-deflection curve. *E_el_* is the elastic energy, determined as the elastic recoverable energy upon unloading at failure, which represented by the triangular shaded area in Figure 11, with the unloading slope estimated by the initial post-cracking stiffness (S).

**Figure 12:**
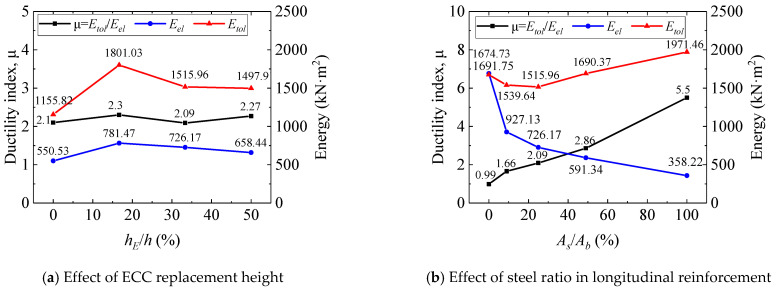
The ductility index.

Results of the energy-based ductility assessment are shown in Figure 12. Figure 12a indicates that for identical bottom reinforcement, increasing the ECC replacement height (*h_E_*/*h* from 0% to 50%) increased the ductility index by 8.09%. This improvement arises from ECC’s multiple cracking mechanism; after cracking, the ECC layer continues to carry load via fiber bridging, retarding stiffness degradation and enhancing energy dissipation capacity. Figure 12b reveals the influence of the steel ratio under constant ECC replacement. As the bottom steel ratio (*A_s_*/*A_b_*) increased, the elastic energy (*E_el_*) gradually decreased, while the total failure energy (*E_tol_*) decreased initially before increasing, resulting in a progressive increase in the ductility index (*μ*). This trend stems from a shift in the material’s energy transformation mechanism: elastic energy is primarily provided by the GFRP layer and the steel core before yielding, hence lower steel ratios lead to higher *E_el_* Plastic energy depends on the post-yield plastic deformation of the steel core; above a steel ratio of approximately 25%, the incremental plastic energy compensates for the reduction in elastic energy, causing *E_tol_* to first decrease then increase. The overall trend confirms that higher bottom steel ratios enhance ductility. Therefore, SFCBs with an appropriate steel ratio offer a balance, ensuring adequate elastic energy reserves while utilizing plastic deformation to improve structural failure toughness. It should be noted that the ductility index (*μ*) calculated based on Equation (1) exhibits a linear relationship with the unloading stiffness, S. However, the method for estimating *S* based on conventional concrete beams, and its applicability to ECC-concrete composite beams requires further validation in subsequent research.

Furthermore, the results in Figure 12 indicate a non-linear relationship between the steel ratio and the ductility index. While the ductility index (*μ*) increases with the steel ratio, a deeper analysis reveals a trade-off between different performance metrics. Specimens with a very high steel ratio (e.g., B-S20-E90, 100%) exhibited superior crack control and higher flexural stiffness prior to yielding. Conversely, specimens with a moderately high steel ratio (e.g., B-S14G3-E90, 49%) demonstrated a more pronounced strain-hardening behavior after yielding, leading to the development of a denser micro-crack network (as observed in Figure 10) and consequently, higher energy dissipation capacity. This suggests that for practical design, an intermediate steel ratio in the range of 25% to 49%may offer the optimum balance, ensuring adequate serviceability performance (stiffness and crack control) while maximizing structural toughness and ductility at the ultimate limit state.

### 3.6. Preliminary Economic Analysis

This section provides a preliminary economic assessment of the SFCB-reinforced ECC-concrete composite beams, focusing on the material cost-effectiveness based on current market prices in China. The unit costs of raw materials for fabricating the ECC are listed in Table 6. The resultant material cost of ECC is calculated to be 4458.84 RMB/m^3^, compared to 350 RMB/m^3^ for conventional C40 concrete. The unit price of steel rebar is 4000 RMB/ton, and the unit price of GFRP bar is 12,000 RMB/ton. As the manufacturer of the SFCBs did not provide a separate fabrication cost, the unit cost of the SFCBs was estimated based on the proportional material costs of their steel core and GFRP sheath.

**Figure 13:**
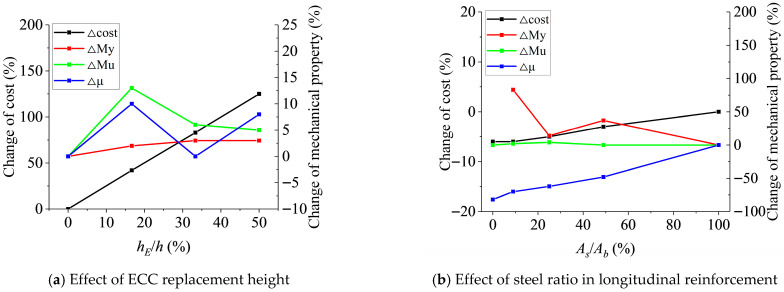
Cost–benefit analysis.

Although a comprehensive life-cycle cost analysis is beyond the scope of this paper, a preliminary evaluation was conducted based on material costs and experimental results, as illustrated in Figure 13. Figure 13a shows the incremental cost and mechanical performance improvements under different ECC replacement ratios. It can be observed that the incremental cost of the composite beams increases linearly with a higher ECC replacement ratio, while the improvements in ultimate bending capacity and ductility index first increase and then decrease. Notably, with only a modest increase in material cost, a replacement ratio of 16.67% yields the most significant performance gain, enhancing the ultimate moment capacity by 12.86% and the ductility index by 9.52%. Figure 13b presents the economic analysis concerning the steel ratio in the bottom longitudinal reinforcement. Compared to the pure steel-reinforced ECC-concrete composite beam, the cost of the SFCB-reinforced beam decreases as the steel ratio decreases. This is because, although the unit price of GFRP is higher than that of steel, its density is significantly lower. A steel ratio of 50% was found to offer the most favorable short-term economic benefit. Compared to the pure steel-reinforced beam, the SFCB-reinforced beam with a 50% steel ratio exhibits a −3.09% cost increment (indicating cost savings), a 37.43% increase in yield capacity, and a less than 50% decrease in ductility. This highlights the potential of SFCBs for optimizing the economic and mechanical performance of composite structures.

Obviously, ECC incurs high costs in short-term use—primarily due to PE fibers. In subsequent research, cost reduction can be achieved by exploring methods to reduce the dosage of PE fibers or substituting them with alternative fibers. Furthermore, ECC’s characteristic multiple micro-cracking behavior allows it to effectively block the ingress of erosive substances compared to conventional concrete, thereby enhancing structural durability and reducing long-term maintenance costs. Although a comprehensive life-cycle cost analysis is beyond the scope of this paper, this study provides a crucial foundation for evaluating the economic feasibility of this composite technology.

## 4. Conclusions

Four-point bending tests were conducted on eight SFCB-reinforced ECC-concrete composite beams to investigate the effects of ECC replacement height and bottom steel ratio on failure modes, crack propagation, load-carrying capacity, deformation capacity, and ductility. The main conclusions are as follows:

(1) All composite beams failed in an under-reinforced flexural mode governed by concrete crushing. Beams with steel/SFCB reinforcement failed after yielding of the reinforcement followed by concrete crushing, while the GFRP-reinforced beam failed by crushing without reinforcement rupture. ECC replacement significantly increased micro-crack density in the tension zone. However, failure modes were highly similar across different reinforcement types, confirming a stable synergistic interaction between ECC and reinforcement.
(2) ECC replacement had a negligible influence on flexural stiffness but enhanced both ultimate flexural capacity (*M_u_*) and ductility. For identical bottom reinforcement, increasing the ECC replacement ratio (*h_E_*/*h*) from 0% to 50% increased Mu and the ductility index (*μ*) by 4.79% and 8.09%, respectively.
(3) Under a constant total reinforcement ratio, the bottom steel ratio (*A_s_*/*A_b_*) significantly governed the structural response. Higher steel ratios increased pre-yield flexural stiffness and yield moments, providing better crack control during the serviceability phase. Importantly, an intermediate steel ratio range of 25% to 49% was found to provide an optimal balance, offering excellent post-yield ductility and energy dissipation capacity through distributed cracking, while maintaining satisfactory initial stiffness. A 100% steel ratio (pure steel) is not necessary to achieve superior ductile performance.
(4) The ECC layer dispersed macro-cracks into micro-cracks via fiber bridging, delaying stiffness degradation and enhancing energy dissipation capacity. However, the reinforcement remained the dominant element for tension resistance irrespective of ECC inclusion. Increasing the bottom steel ratio decreased the elastic energy (*E_el_*) while increasing the plastic energy component, leading to overall higher ductility. Design should therefore balance the elastic/plastic energy distribution to ensure both structural strength and toughness.
(5) Limitations and Future Work: This study provides foundational experimental data on the flexural behavior of SFCB-ECC-concrete composite beams. The findings demonstrate promising synergy, but their generalization for direct design application requires further development. Future work will focus on two key areas: (i) Theoretical Modeling: Utilizing the experimental results from this study to develop and validate analytical models and numerical simulations for predicting the structural response, ultimately leading to proposed design guidelines. (ii) Practical Validation: Extending the validation beyond laboratory-scale specimens through large-scale or full-scale tests and investigating the long-term performance under environmental exposure and cyclic loading to fully assess the technology’s real-world applicability. (iii) This study demonstrates robust composite action in SFCB-ECC-concrete beams under flexure, with no interfacial failure observed. However, deriving specific design parameters (e.g., shear friction coefficients) requires dedicated future research using direct shear or slant shear tests to quantitatively characterize the ECC-concrete interface.

## Figures and Tables

**Table 1:** Mix proportion of ECC (kg/m^3^).

Cement	FA	QP	Water	Superplasticizer	PE Fibers	VMA	Defoamer
600	600	432	348	1.8	19.4	1.8	1.2

**Table 2:** Detailed physical parameters of PE fiber.

Density (g/cm^3^)	Strength (MPa)	Elastic Modulus (GPa)	Length (mm)	Diameter (μm)	Elongation at Break (%)
0.97	2500	120	12	20	3.7

**Table 3:** Mechanical properties of rebars.

No.	*d* (mm)	*d_s_* (mm)	*t_f_* (mm)	*E_I_* (GPa)	*f_y_* (MPa)	*E_II_* (GPa)	*f_u_* (MPa)
G20	20	0	10	46.48	/	/	959.77
S20	20	20	0	174.61	439.6	/	616.88
S6G7	20	6	7	53.52	136.5	42.11	833.44
S10G5	20	10	5	77.73	195.23	29.78	771.75
S14G3	20	14	3	111.49	265.05	13.67	464.76

Note: *d* is the nominal diameter of rebars; *d_s_* is the steel core diameter; *t_f_* is the thickness of outer GFRP layer; *E_I_* is the elastic modulus; *E_II_* is the post-yield hardening modulus; *f_y_* is the yield strength; *f_u_* is the ultimate tensile strength.

**Table 4:** Test piece design parameters.

Specimens	Bottom Longitudinal Reinforcement	*A_s_*/*A_b_* (%)	*h_E_* (mm)	*h_E_*/*h* (%)
B-S10G5-E0	2S10G5	25	0	0.00
B-S10G5-E45	2S10G5	25	45	16.67
B-S10G5-E90	2S10G5	25	90	33.33
B-S10G5-E135	2S10G5	25	135	50.00
B-S6G7-E90	2S6G7	9	90	33.33
B-S14G3-E90	2S14G3	49	90	33.33
B-S20-E90	2S20	100	90	33.33
B-G20-E90	2G20	0	90	33.33

Note: The specimen designation includes three parts. The first part ‘B’ denotes beam. The second part indicates the bottom reinforcement configuration: for example, S10G5 specifies SFCBs with a steel core diameter of 10 mm and an outer GFRP layer thickness of 5 mm. The third part indicates the ECC replacement height: for example, E90 denotes an ECC replacement height of 90 mm. *A_s_* is the cross-sectional area of the steel core within the bottom longitudinal reinforcement, *Ab* is the total cross-sectional area of the bottom longitudinal reinforcement, *h_E_* is the ECC replacement height within the tension zone, *h* is the cross-sectional height of the composite beam.

**Table 5:** Test results of beam specimen.

Specimen	*Δ_cr_* (mm)	*P_cr_* (kN)	*M_cr_* (kN·m)	*Δ_y_* (mm)	*P_y_* (kN)	*M_y_* (kN·m)	*Δ_u_* (mm)	*P_u_* (kN)	*M_u_* (kN·m)
B-S10G5-E0	0.93	17.01	5.95	8.71	102.09	35.73	28.4	185.48	64.92
B-S10G5-E45	0.95	22.17	7.76	9.32	104.31	36.51	38.69	209.34	73.27
B-S10G5-E90	1.28	22.63	7.92	10.15	104.94	36.73	35.06	196.23	68.68
B-S10G5-E135	1.07	21.8	7.63	9.39	105.35	36.87	34.34	194.38	68.03
B-S20-E90	0.63	24.25	8.49	8.54	168.28	58.90	35.46	193.06	67.57
B-S14G3-E90	1.05	23.19	8.12	10.8	126.63	44.32	36.67	189.34	66.27
B-S6G7-E90	1.45	21.95	7.68	11.81	92.15	32.25	38.74	189.47	66.31
B-G20-E90	1.59	22.21	7.77	/	/	/	46.59	189.67	66.38

Note: *Δ_c_*, *P_cr_*, *M_cr_* is the mid-span deflection, load, and bending moment at cracking; *Δ_y_*, *P_y_*, *M_y_* is the mid-span deflection, load, and bending moment at yield; *Δ_u_*, *P_u_*, *M_u_* is the mid-span deflection, load, and bending moment at failure.

**Table 6:** Unit cost of raw materials for fabricating ECC.

Content	Cost (RMB/kg)
Cement	0.7
FA	0.539
QP	0.392
Water	0.002
Superplasticizer	3
PE Fibers	180
VMA	20
Defoamer	10

## Data Availability

The raw data supporting the conclusions of this article will be made available by the authors upon request.
